# Bilateral chylothorax following neck dissection on a child—Diagnosis and management in resource-limited settings: a case report

**DOI:** 10.1093/jscr/rjab600

**Published:** 2022-01-13

**Authors:** Kennedy K Misso, Joseph Elisante, Daniel Mganga, Vanessa Poppe, Murad Tarmohammed, Mugisha Nkoronko, David Msuya

**Affiliations:** Department of General Surgery, Kilimanjaro Christian Medical Centre, Moshi Tanzania; Department of General Surgery, Kilimanjaro Christian Medical University College, Moshi Tanzania; Department of General Surgery, Kilimanjaro Christian Medical Centre, Moshi Tanzania; Department of General Surgery, Kilimanjaro Christian Medical University College, Moshi Tanzania; Department of General Surgery, Kilimanjaro Christian Medical Centre, Moshi Tanzania; Department of General Surgery, Kilimanjaro Christian Medical Centre, Moshi Tanzania; Department of General Surgery, Kilimanjaro Christian Medical University College, Moshi Tanzania; Department of General Surgery, Kilimanjaro Christian Medical Centre, Moshi Tanzania; Department of General Surgery, Kilimanjaro Christian Medical University College, Moshi Tanzania; Department of General Surgery, Kilimanjaro Christian Medical Centre, Moshi Tanzania; Department of General Surgery, Kilimanjaro Christian Medical University College, Moshi Tanzania; Department of General Surgery, Kilimanjaro Christian Medical Centre, Moshi Tanzania; Department of General Surgery, Kilimanjaro Christian Medical University College, Moshi Tanzania

## Abstract

Bilateral chylothorax is a rare complication following neck dissection, with fewer than thirty cases being reported over the last century. A serious life-threatening condition mostly encountered during thoracic procedures and dissections. In our case, conservative management resulted in complete resolution. We report a case of a 4-year-old child who underwent deep neck dissection due to recurrent hemangioma. She developed bilateral chylothorax and a conservative approach led to complete resolution.

## INTRODUCTION

Chyle has milky appearance with fat levels ranging from 0.4 to 4%, depending on diet. Chyle has no odor with variable composition in leukocyte count and 40–80% albumin [1].

The thoracic duct drains all body lymph with the exception of the right side of the body above the diaphragm. The duct lies inside the visceral compartment posterior to the carotid sheath. The location of the termination within the neck is highly variable. With variations accounting for most of the injuries, termination of the left internal jugular vein is the most common site of iatrogenic injury [1, 2].

Chylothorax has serious complications due to metabolic and physiological derangements. Mechanical complications arise from accumulation of chyle within pleural spaces with limited vital capacity and compression of the mediastinal structures and major vessel distortion [2, 3]. Stuart reviewed 40 cases in 1907 of thoracic duct injury in the neck. Three cases had bilateral chylothorax.

Chylothorax etiology can be grouped into three categories: congenital, traumatic and obstructive. Traumatic chylothorax is uncommon following neck dissections. Neither the less bilateral nor the more unilateral chylothorax is well known [4]. Obstructive chylothoraxes can be intraluminal or extraluminal, from infectious or neoplastic causes [4].

## CASE REPORT

**Figure 1:**
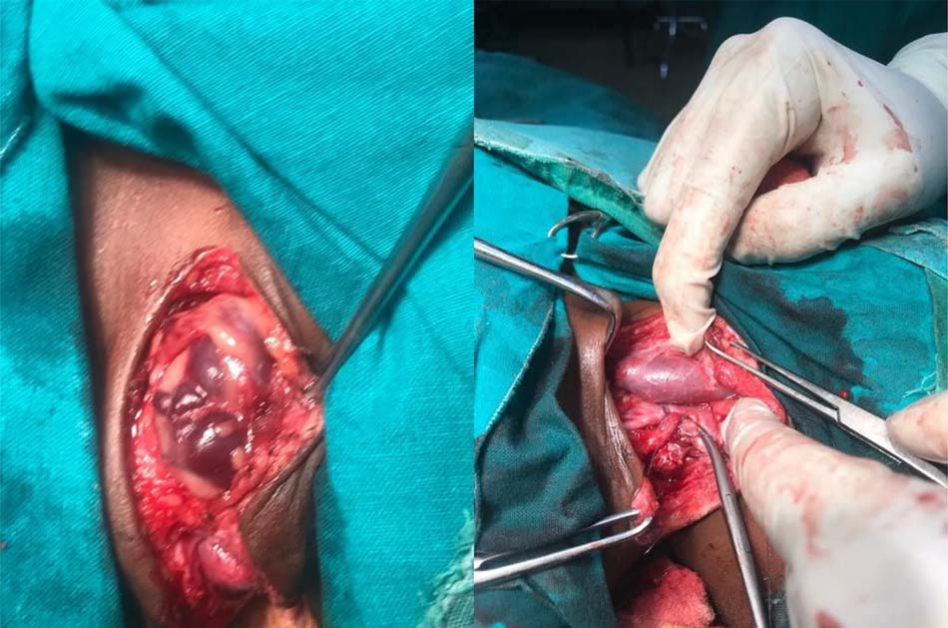
Lesion within the neck, before (left) and after dissection.

A report of a 4-year-old female referred to our facility with a recurrent, painless neck swelling on the left side for 2 years is presented. She underwent excision of the swelling twice over a period of 2 years with recurrences. Clinically, the lesion was in the posterior triangle, 4 by 5 cm, painless, firm with no color change and no bruits upon auscultation. She was then scheduled for excisional biopsy. Intraoperatively, the lesion was found to extend deep adjacent to the carotid sheath (Fig. 1). The intraoperative diagnosis was hemangioma.

**Figure 2:**
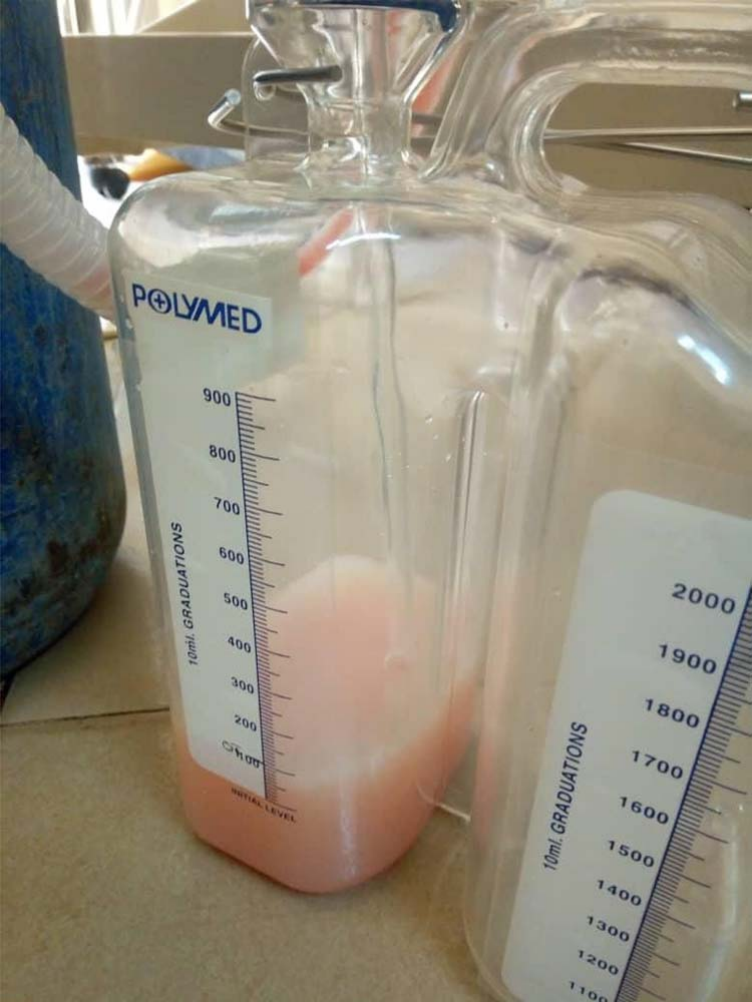
Collection chamber filled with 100 ml of chyle.

Dissection of the lesion was done with no major damage to the neurovascular or respiratory structures. The thoracic duct was injured, identified and ligated. The patient recovered well post-anesthesia and surgery, and was sent to surgical pediatric ward. At 18 hours post-surgery, she had progressive difficulty breathing, necessitating oxygen support through nasal prongs. Respiratory examination revealed reduced air entry bilaterally. Diagnostic needle thoracostomy was done, and it revealed milk-like fluid bilaterally. Chest tubes inserted bilaterally and drained a total of 250 ml of chyle (150 ml and 100ml), the latter being on the right side (Fig. 2). Drained fluid had higher triglyceride levels (130 mg/dl) than serum levels (80 mg/dl).

**Figure 3:**
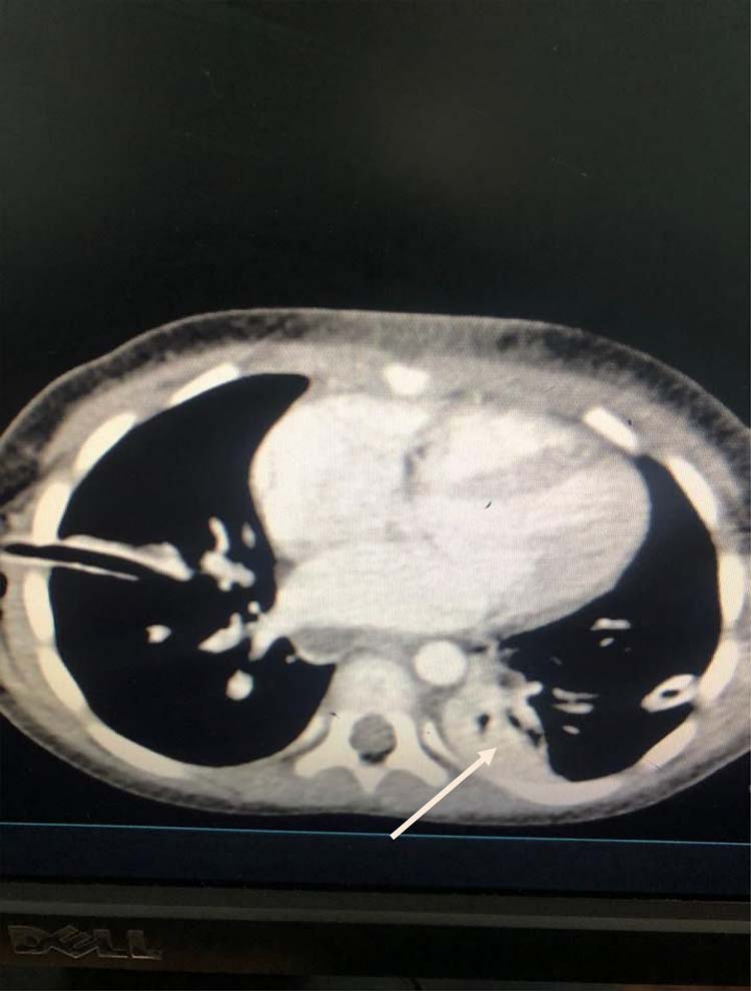
Axial Slice of the thorax showing bilateral thoracostomy tubes and minimal fluid on the left side (white arrow).

She was kept on conservative management with lipid restriction and octreotide. A day later, she had resolved respiratory signs and symptoms. Control computed tomography (CT) revealed significant improvements with minimal fluid on the left side (Fig. 3).

**Figure 4:**
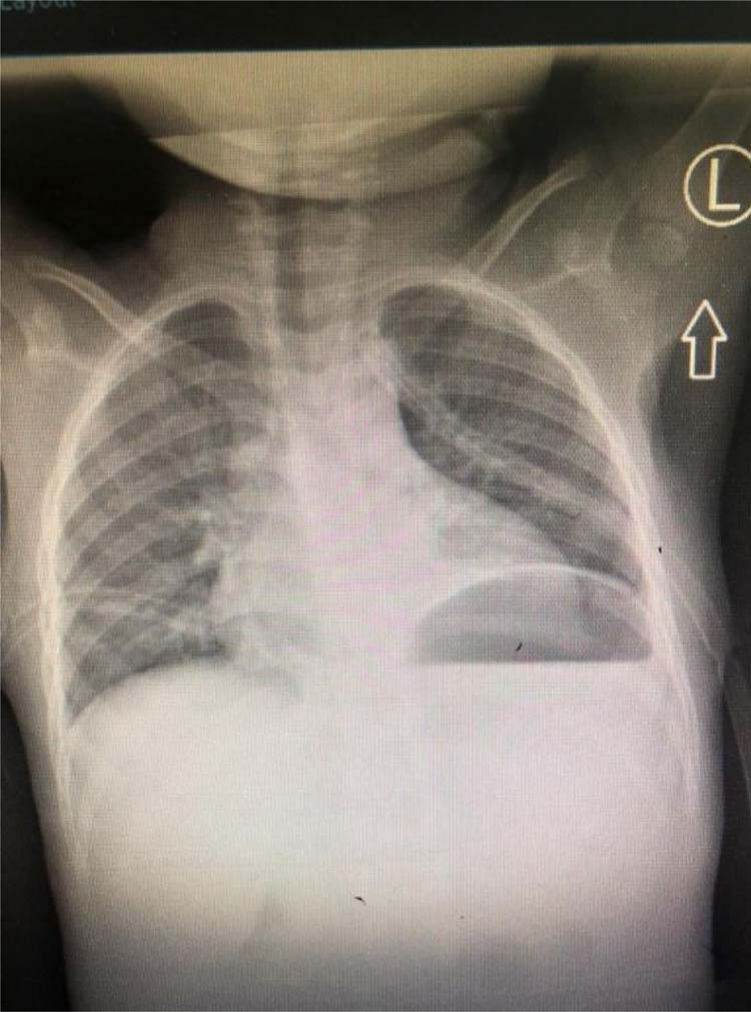
Control Chest X-ray showing resolution of the chylothorax with thoracostomy tubes *in situ*.

A total of 48 hours post tube thoracostomy patient had no drainage with complete resolution upon control X-ray (Fig. 4). She was then discharged and attended our outpatient department. She had an optimal recovery.

## DISCUSSION

Diagnosis of chylothorax relies on clinical, radiological and biochemical confirmation of the chyle. Sudan III Stain has high fat globules affinity and may aid in establishing the diagnosis. Other methods include higher triglyceride’s measurement in chyle in comparison with plasma levels and lipoprotein electrophoresis [5]. Our patient had vivid milky fluid that had higher levels of triglycerides in comparison with plasma levels. Chest CT revealed minimal effusions following tube thoracostomies.

The exact mechanism leading to chylothorax is yet to be fully understood. Two possible mechanisms can explain the plausibility of chylothorax, chyle extravasating from the neck may track into mediastinum and be retained, followed by negative intrathoracic pressure to cause chylothorax [6]. Second hypothesis is ligation of the thoracic duct leads to congestion of the friable intrathoracic thoracic duct, with constant intrathoracic pressure causes it to drain into mediastinal and pleural spaces [7]. The later mechanism could shade light in regard to our case. Child had a drain in the neck region that was draining serous fluid. Thoracic duct was injured, identified and ligated. Chyle backflow caused a buildup in hydrostatic pressure, with an increase in pressure and volume above the collateral capacity possibly due to underdevelopment in regard to our patient’sage.

Management of chylothorax is mostly conservative. With drainage of chyle from pleural cavity, minimizing chyle production and optimal nutrition support [4]. Somatostatin analogs decrease splanchnic blood flow, and in animals it has been shown to lessen thoracic duct lymph flow [5, 8]. Among reported cases of bilateral chylothorax, none involved a child. Our patient was well managed with the aforementioned approach, stayed with chest drains for ~3 days with optimal recovery and discharge within a week, outpatient visits revealed an otherwise healthy child. Few cases have been reported to require thoracic duct ligation, intra-abdominal or thoracic approaches Indications comprise malnutrition and metabolic complications and persistence leakage for 2 weeks [5].

## CONCLUSION

Chylothoraxes is a rare complication following neck dissections. Carrying significant morbidity and mortality when is not fully attended. Current recommendations include conservative approach as the first line of management [9]. Early diagnosis and management bear a vital role in the prevention of serious cardiorespiratory, nutritional and metabolic derangements.

## CONFLICT OF INTEREST STATEMENT

None declared.

## FUNDING

None.
